# Human *decidua basa*lis mesenchymal stem/stromal cells protect endothelial cell functions from oxidative stress induced by hydrogen peroxide and monocytes

**DOI:** 10.1186/s13287-018-1021-z

**Published:** 2018-10-25

**Authors:** M. A. Alshabibi, T. Khatlani, F. M. Abomaray, A. S. AlAskar, B. Kalionis, S. A. Messaoudi, R. Khanabdali, A. O. Alawad, M. H. Abumaree

**Affiliations:** 1 National Center for Stem Cell Technology, Life Sciences and Environment Research Institute, King Abdulaziz City for Science and Technology, P.O Box 6086, Riyadh, 11442 Kingdom of Saudi Arabia; 2 Stem Cells and Regenerative Medicine Department, King Abdullah International Medical Research Center, King Abdulaziz Medical City, Ministry of National Guard Health Affairs, Mail Code 1515, P.O. Box 22490, Riyadh, 11426 Saudi Arabia; 3 Division of Obstetrics and Gynecology, Department of Clinical Science, Intervention and Technology, Karolinska Institutet, 141 86 Stockholm, Sweden; 4 Center for Hematology and Regenerative Medicine, Karolinska Institutet, 141 86 Stockholm, Sweden; 5 College of Medicine, King Saud Bin Abdulaziz University for Health Sciences, King Abdulaziz Medical City, Ministry of National Guard Health Affairs, Mail Code 3124, P.O. Box 3660, Riyadh, 11481 Saudi Arabia; 6 Adult Hematology and Stem Cell Transplantation, King Abdulaziz Medical City, Ministry of National Guard Health Affairs, Mail Code 1515, P.O. Box 22490, Riyadh, 11426 Saudi Arabia; 7 Department of Maternal-Fetal Medicine Pregnancy Research Centre, University of Melbourne, Melbourne, Australia; 8 Department of Obstetrics and Gynaecology, Royal Women’s Hospital, Parkville, Melbourne, Victoria 3052 Australia; 9 Department of Forensic Biology, College of Forensic Sciences, Naif Arab University for Security Sciences, Riyadh, Saudi Arabia; 10 College of Science and Health Professions, King Saud Bin Abdulaziz University for Health Sciences, King Abdulaziz Medical City, Ministry of National Guard Health Affairs, Mail Code 3124, P.O. Box 3660, Riyadh, 11481 Saudi Arabia

**Keywords:** Placenta, *Decidua basalis* mesenchymal stem cells, Endothelial cells, H_2_O_2_, Proliferation, Adhesion, Migration, Monocytes

## Abstract

**Background:**

Human *decidua basalis* mesenchymal stem/multipotent stromal cells (DBMSCs) inhibit endothelial cell activation by inflammation induced by monocytes. This property makes them a promising candidate for cell-based therapy to treat inflammatory diseases, such as atherosclerosis. This study was performed to examine the ability of DBMSCs to protect endothelial cell functions from the damaging effects resulting from exposure to oxidatively stress environment induced by H_2_O_2_ and monocytes.

**Methods:**

DBMSCs were co-cultured with endothelial cells isolated from human umbilical cord veins in the presence of H_2_O_2_ and monocytes, and various functions of endothelial cell were then determined. The effect of DBMSCs on monocyte adhesion to endothelial cells in the presence of H_2_O_2_ was also examined. In addition, the effect of DBMSCs on HUVEC gene expression under the influence of H_2_O_2_ was also determined.

**Results:**

DBMSCs reversed the effect of H_2_O_2_ on endothelial cell functions. In addition, DBMSCs reduced monocyte adhesion to endothelial cells and also reduced the stimulatory effect of monocytes on endothelial cell proliferation in the presence of H_2_O_2_. Moreover, DBMSCs modified the expression of many genes mediating important endothelial cell functions. Finally, DBMSCs increased the activities of glutathione and thioredoxin reductases in H_2_O_2_-treated endothelial cells.

**Conclusions:**

We conclude that DBMSCs have potential for therapeutic application in inflammatory diseases, such as atherosclerosis by protecting endothelial cells from oxidative stress damage. However, more studies are needed to elucidate this further.

## Background

Mesenchymal stem cells (MSCs) are adult multipotent stromal cells that can be isolated from many tissues, such as human placenta [1]. Recently, we isolated MSCs from the maternal *decidua basalis* tissue (DBMSCs) of human term placenta [2]. The tissue of *decidua basalis* is a main source of oxidative stress molecules, which are found in the maternal circulation due to pregnancy [3]. Therefore, DBMSCs in their niche (vascular microenvironment) are in direct contact with the maternal circulation, and therefore, they are exposed to high levels of inflammation and oxidative stress mediators [4]. In addition, we also isolated MSCs from the fetal tissue (chorionic villi) of the placenta [5]. These fetal chorionic MSCs are in direct contact with the fetal circulation and therefore exposed to lower levels of inflammation and oxidative stress molecules as compared to DBMSCs [5–7].

MSCs from placenta and other sources can differentiate into multiple cell lineages including adipocyte, osteoblast, and chondrocyte [1]. In addition, MSCs show low immunogenicity and anti-inflammatory properties [1]. Therefore, MSCs have been investigated as promising therapeutic agents in many inflammatory diseases, such as atherosclerosis [8].

Atherosclerosis is characterized by endothelial activation due to the accumulation of high amounts of low-density lipoprotein (LDL) and immune cells that lead to the production of high levels of oxidative stress mediators, such as hydrogen peroxide (H_2_O_2_) [9, 10].

H_2_O_2_ has several important effects on endothelial cell functions in physiological homeostasis and in inflammatory diseases [9, 10]. H_2_O_2_ alters the functional activities of proteins that cause the generation of more toxic radicals (i.e., peroxynitrite (ONOO^−^) and hydroxyl (·OH)), which induce oxidative damage in the cellular DNA and proteins [9, 10]. In addition, H_2_O_2_ can rapidly inactivate nitric oxide (NO) and this causes endothelial cell damage [9, 10].

Endothelial cell damage is usually associated with phenotypic changes (i.e., increased expression of inflammatory molecules), dysfunctional activities [i.e., increased endothelial cell proliferation, adhesion, migration, permeability, angiogenesis (blood vessel formational)], and also enhanced endothelial cell interaction with immune cells (i.e., enhanced monocyte adhesion to the endothelium and their infiltration into the tissues); these events are the typical characteristics of atherosclerosis [11]. In atherosclerosis, an inflammatory response is initiated at the injury site of endothelium that increases the expression of adhesion molecules (i.e., VCAM-1), which activates the recruitment and adhesion of immune cells (i.e., monocytes) to the injured site of endothelium [11]. This interaction between monocytes and endothelial cells will loosen up the tight junction between endothelial cells that increases the permeability of endothelium and subsequently monocytes and LDL will pass through the intima, where LDL undergoes oxidation while monocytes differentiate into macrophages, which take up oxidized LDL [11]. This lipid laden macrophages are known as “foam cells”, which eventually die by apoptosis, but the lipid content will accumulate in the intimal area leading to the formation of plaque [11].

Recently, we reported that DBMSCs can protect endothelial cells from activation by inflammation triggered by monocyte adhesion and increased endothelial cell proliferation [12]. These events are manifest in inflammatory diseases, such as atherosclerosis. These data make DBMSCs as a useful candidate to be employed in a therapeutic strategy for treating atherosclerosis. We performed this study to examine the ability of DBMSCs to protect endothelial cell functions from the damaging effects resulting from exposure to oxidatively stress environment induced by H_2_O_2_ and monocytes. We investigated the ability of DBMSCs to protect endothelial cell functions (adhesion, proliferation, and migration) from oxidative stress induced by H_2_O_2._ The effect of DBMSCs on the adhesion of monocytes to endothelial cells in oxidative stress environment was also examined. Finally, we investigated the effect of DBMSCs on endothelial cell expression of many genes under oxidative stress, and the mechanism underlying DBMSC protection of endothelial cells from oxidative stress was also determined. Our data suggest that DBMSCs have a protective effect on endothelial cells in oxidative stress environment and suggest that DBMSCs have the potential to treat inflammatory diseases, such as atherosclerosis by protecting endothelial cells from injury induced by oxidative stress and inflammatory cells. However, future studies are necessary to elucidate this further in vitro and in vivo.

## Methods

### Ethics and collection of human placentae and umbilical cords

The study was approved by the institutional review board (reference number IRBC/246/13) of KAIMRC (King Abdulla International Medical Research Centre, Saudi Arabia). Samples (placentae and umbilical cords of uncomplicated human pregnancies, 38–40 gestational weeks) were obtained and used immediately after signing consent forms. All clinical and experimental procedures were performed in compliance with KAIMRC research guidelines and regulations.

### Isolation and culture of DBMSCs

MSCs were isolated from the *decidua basalis* (DBMSCs) of the maternal part of human term placenta as previously described by us [2]. Briefly, the decidual tissues were dissected and then digested using a sterile phosphate-buffered solution (PBS; pH 7.4) containing 0.3% collagenase type I (Life Technology, Grand Island, USA), 270 unit/mL DNase I (Life Technology), and antibiotics (100 μg/mL streptomycin and 100 U/mL penicillin). After 1-h incubation at 37 °C in a water bath, the cell mixture was filtered through a 100-μm nylon filter (Becton Dickinson, NJ, USA), and the red blood cells in the cell pellet were then removed as previously described [12]. Cells were then washed with sterile PBS and cultured in a complete DBMSC culture medium [DMEM-F12 medium containing 10% MSCFBS (mesenchymal stem cell-certified fetal bovine serum, catalogue number 12-662-011, Life Technology), and antibiotics described above] and then incubated at 37 °C in a humidified atmosphere containing 5% CO_2_ and 95% air (a cell culture incubator). Prior to using DBMSCs in subsequent experiments, DBMSCs at passage 3 were characterized by flow cytometry using MSC and hematopoietic markers (Table 1) and then evaluated for differentiation into adipocytes, chondrocytes, and osteocytes as previously described by us [2]. DBMSCs (passage 3) of 30 placentae were used in this study.

**Table 1:** Monoclonal antibodies used in this study

Markers	Monoclonal antibodies							
MSC markers	CD44	CD90	CD105	CD146	CD166	HLA-ABC		
Hematopoietic markers	CD14	CD19	CD40	CD45	CD80	CD83	CD86	HLA-DR
Endothelial Cell Marker	CD31							
Adhesion Molecules	ICAM-1	VCAM-1	CD44					

### Isolation and culture of human umbilical vein endothelial cells (HUVEC)

HUVEC were isolated from umbilical cord veins using our previously published method [12]. Following rinsing the cannulated umbilical veins with PBS for several times, veins were filled with a digestion PBS solution containing 6 mg/ml collagenase type II (catalogue number 17101-015, Life Technologies) and then incubated at 37 °C in a cell culture incubator. After 25 min, HUVEC were collected and then resuspended in a complete endothelial cell growth medium (catalogue number PCS-100-041™, ATCC, USA) and cultured at 37 °C in a cell culture incubator as previously described [12]. Prior to using HUVEC in subsequent experiments, they were characterized by flow cytometry using a CD31 endothelial cell marker (R and D Systems, Abingdon, UK) as previously described [12]. HUVEC (> 95% purity) from passages 3 to 5 of 30 umbilical cords were used in this study.

### HUVEC proliferation in response to DBMSCs and H_2_O_2_

HUVEC (5 × 10^3^) were seeded in wells of 96-well culture plates containing a complete endothelial cell growth medium and cultured at 37 °C in a cell culture incubator. Following 24 h, adherent HUVEC were incubated with different concentrations [1%, 5% and 25% (*v*/*v*) conditioned medium (CM) harvested from DBMSC culture (CMDBMSC) diluted in a complete DBMSC growth medium] of CMDBMSC and different ratios of 1:1, 5:1, and 10:1 HUVEC to DBMSC. Cells were then cultured in a complete endothelial cell growth medium with or without 100 μM H_2_O_2_ for 72 h at 37 °C in a cell culture incubator.

HUVEC proliferation was then evaluated after each indicated culture time points (24, 48, and 72 h) by a tetrazolium compound [3-(4,5-dimethylthiazol-2-yl)-5-(3-carboxymethoxyphenyl)-2-(4-sulfophenyl)-2H-tetrazolium, inner salt; MTS] kit (catalogue number G5421, CellTiter 96® Aqueous Non-Radioactive Cell Proliferation Assay, Promega, Germany), as previously described [12]. CMDBMSC was produced as previously described [12]. Before adding DBMSCs to HUVEC culture, DBMSCs were treated with 25 μg/ml Mitomycin C to inhibit their proliferation as previously described [12]. The blank was cells incubated in MTS solution in a complete endothelial cell growth medium alone. Results were presented as means (± standard errors). Each experiment was performed in triplicate and repeated with five independent HUVEC (passages 3–5) and DBMSC (passage 3) preparations.

### Culture of HUVEC with different treatments of DBMSCs (conditioned medium, supernatant, and intercellular direct contact) and H_2_O_2_

**Fig. 1:**
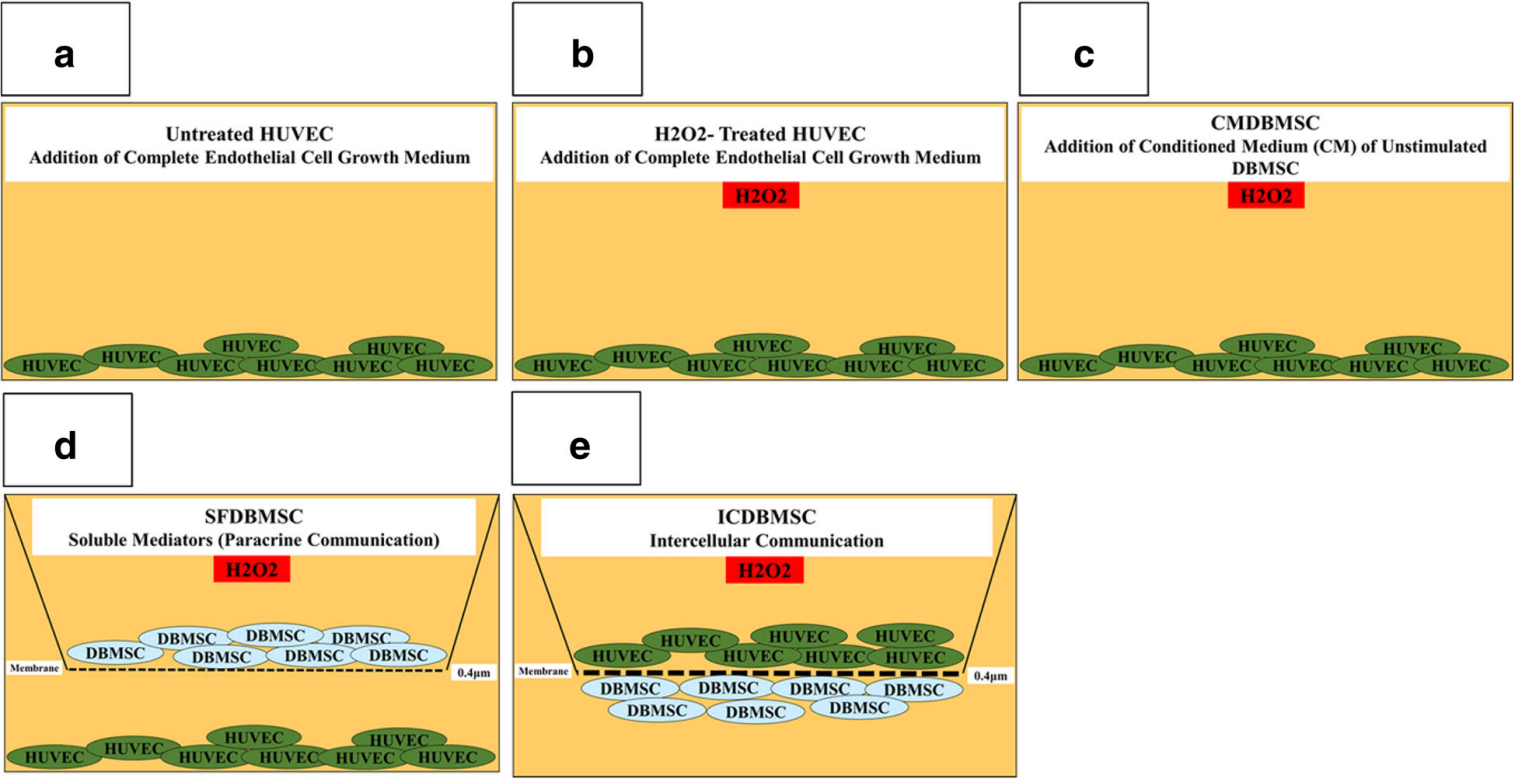
The culture system used in this study to culture HUVEC alone or with H_2_O_2_ in the presence or absence of different treatments of DBMSCs (CMDBMSC, SFDBMEC, and ICDBMSC). CMDBMSC culture system consisted of HUVEC seeded on a surface of 6-well culture plate in a complete endothelial cell growth culture medium (untreated HUVEC) (**a**) or with 100 μM H_2_O_2_ (**b**) or with 100 μM H_2_O_2_ and 25% CM obtained from unstimulated DBMSCs (**c**); SFDBMSC culture system consisted of DBMSCs seeded in the upper chamber while HUVEC seeded in the lower chamber of transwell membrane culture system (**d**); and ICDBMSC culture system consisted of DBMSCs seeded on the reverse side of the membrane of the chamber and HUVEC seeded on the upper side of the membrane (**e**). For SFDBMSC and ICDBMSC, 0.4-μm pore size transwell chamber membrane was used. HUVEC were incubated with different concentrations (1%, 5%, and 25% (*v*/*v*) CM diluted in complete DBMSC growth medium) of CMDBMSC and different ratios of 1:1, 5:1, and 10:1 HUVEC:DBMSC. Cells were then cultured in a complete endothelial cell growth medium with or without 100 μM H_2_O_2_ for 72 h at 37 °C in a cell culture incubator

HUVEC were cultured alone (Fig. 1a) or with 100 μM H_2_O_2_ (Fig. 1b) or with 25% CMDBMSC and 100 μM H_2_O_2_ (Fig. 1c) in a complete endothelial cell growth medium. For the coculture experiments (supernatant and intercellular direct contact), cells (HUVEC and DBMSCs) were separated by transwell chamber membrane culture system [catalogue number 657640, ThinCert™ Cell Culture Inserts, 0.4 μm, Greiner Bio-One, Germany]. For soluble factor experiments (SFDBMSC; Fig. 1d), DBMSCs were cultured on the upper compartments while HUVEC were cultured in the lower compartment. For intercellular direct contact experiments (ICDBMSC; Fig. 1e), DBMSCs were seeded on the reverse side of the membrane of the chamber, and HUVEC were seeded on the upper side of the membrane. In both culture systems, cells were cultured at 5HUVEC:1DBMSC ratio. Cells in the SFDBMSC and ICDBMSC culture systems were then cultured in a complete endothelial cell growth medium in the presence of 100 μM H_2_O_2_ and incubated as described above. HUVEC were also cultured with CMDBMSC, SFDBMSC, and ICDBMSC without H_2_O_2_. After 48 h in culture, HUVEC were harvested with TrypLE™ Express detachment solution (Life Technologies) and used in an adhesion, proliferation, and migration experiments as described below. HUVEC viability was determined using Trypan blue. Each experiment was performed and repeated as described above. HUVEC cultured in complete endothelial cell growth medium without DBMSCs were included as a negative control for all HUVEC cultured with different treatments of DBMSCs.

### HUVEC adhesion and proliferation using xCELLigence system

The xCELLigence system (RTCA-DP version; Roche Diagnostics, Mannheim, Germany) was used as we previously described [12, 13] to evaluate the adhesion and proliferation of HUVEC. The xCELLigence system is a real-time cell analyzer that constantly monitors and records the changes in electrical impedance, because of cellular events, and these changes are reported as an arbitrary cell index [12, 13]. Briefly, 100-μL complete endothelial cell growth medium was added to well in 16-well culture plates (catalogue number 05469813001, E-Plate 16, Roche Diagnostics), and the background impedance was then achieved as previously described [12, 13]. Then, 20 × 10^4^ HUVEC (HUVEC were initially co-cultured with DBMSCs and 100 μM H_2_O_2_ or cultured alone as described above) were seeded in 100 μL of complete endothelial cell growth medium in quadruplicate wells, and equilibrium was achieved by leaving the culture plates for 30 min at RT before data recording. To record data, culture plates were placed in the xCELLigence system at 37 °C in a cell culture incubator. HUVEC cell index was then automatically monitored for 72 h. For data analysis, the xCELLigence software (version 1.2.1) was used. For cell adhesion, data was measured after 2 h and the value of cell index was then expressed as mean ± standard errors of the cell index. For cell proliferation, data was expressed as mean ± standard errors of the cell index normalized to the cell index recorded after 2 h (adhesion time point). The rate of cell proliferation was determined by calculating the normalized cell index at 24, 48, and 72 h. Each experiment was performed and repeated as described above.

### HUVEC migration using xCELLigence system

The migration of HUVEC was evaluated using CIM migration plates (catalogue number 05665825001, Roche Diagnostics) in the xCELLigence system as previously described by us [12, 13]. The CIM plates have 16-migration wells that each consists of two chambers (upper and lower) separated by a membrane (polyethylene terephthalate) with a porous of 8 μm in size. The membrane is in contact with microelectrodes. Following the addition of 50-μl pre-warmed media to the wells of the upper chamber and 160-μl endothelial cell growth medium containing 30% FBS to the lower chamber, the plates were then locked in the RTCA DP device at 37 °C in a cell culture incubator for 1 h to obtain equilibrium, and a measurement step was then performed as previously described [12, 13]. The migration experiments were then initiated by seeding 20 × 10^3^ HUVEC [HUVEC were initially co-cultured with DBMSCs and 100 μM H_2_O_2_ or cultured with DBMSCs (CMDBMSC, SFDBMSC and ICDBMSC) or cultured alone as described above] in the upper chamber containing 100-μL endothelial cell serum free medium and the plates were then incubated for 30 min at RT to allow the cells to settle onto the membrane as previously described [12, 13]. Experiments were performed in quadruplicate, and after equilibration, the impedance value of each well was automatically monitored every 15 min for 24 h by the xCELLigence system and then expressed as a cell index value. HUVEC migration observed in the presence and absence of 30% FBS served as positive and negative controls, respectively. Each experiment was performed and repeated as described above.

### HUVEC proliferation in response to monocytes pretreated with DBMSCs and H_2_O_2_

**Fig. 2:**
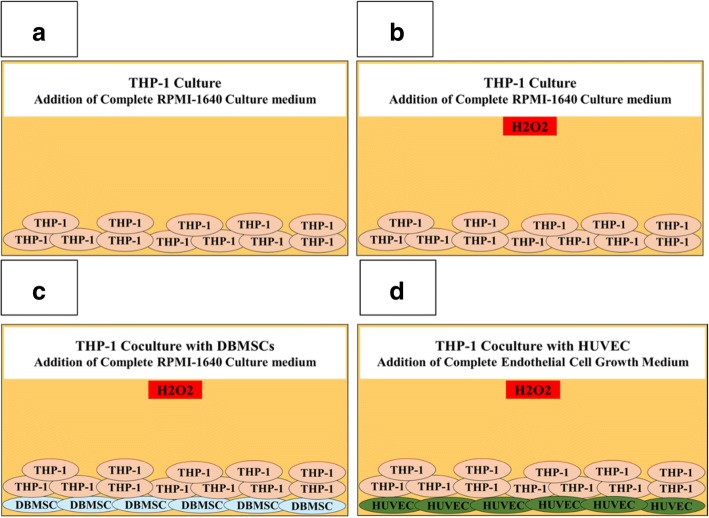
The culture systems used in this study to culture monocytes (THP-1) alone or with H_2_O_2_ or with DBMSCs and H_2_O_2_ or with HUVEC and H_2_O_2_. Monocytes (THP-1) cultured alone in RPMI-1640 culture medium (**a**) or with 100 μM H_2_O_2_ (**b**) or with DBMSCs (physical contact experiment) and 100 μM H_2_O_2_ (**c**), HUVEC cultured with THP-1 pretreated with DBMSCs and 100 μM H_2_O_2_ (Physical contact experiment) in a complete endothelial cell growth medium in the presence of 100 μM H_2_O_2_ (**d**). THP-1 were cultured with DBMSCs at different THP-1:DBMSC ratios (2.5:1, 5:1, 10:1 and 20:1 THP-1:DBMSC) in the presence or absence of 100 μM H_2_O_2_. HUVEC were cultured with THP-1- pre-treated with DBMSCs and H_2_O_2_ at different THP-1:HUVEC ratios (2.5:1, 5:1, 10:1 and 20:1 THP-1:HUVEC) in the presence or absence of 100 μM H_2_O_2_. Cells were then incubated for 96 h at 37 °C in a cell culture incubator

To evaluate the effects of monocytes pre-treated with DBMSCs on the proliferation of endothelial cells, monocyte proliferation in response to DBMSCs was initially examined by adding DBMSCs to human monocytes (THP-1, catalogue number TIB-202™, ATCC, USA) in 96-well tissue culture plates at different THP-1:DBMSC ratios (2.5:1, 5:1, 10:1, and 20:1 THP-1:DBMSC) in the presence or absence of 100 μM H_2_O_2_ (Fig. 2). Cells were then cultured in a complete RPMI-1640 culture medium containing 10% FBS, 100 μg/mL l-glutamate, and antibiotics. After 24, 48, and 72 h incubation at 37 °C in a cell culture incubator, monocyte proliferation was examined using the MTS assay as previously described [12].

Next, endothelial cell proliferation in response to monocytes pre-cultured with DBMSCs and H_2_O_2_ at the indicated ratios (below) in the presence of 100 μM H_2_O_2_ was examined (Fig. 2). After 24-h culture with DBMSCs in the presence of 100 μM H_2_O_2_, THP-1 [THP-1 alone, THP-1+ H_2_O_2_ (THP-1 pretreated with H_2_O_2_), and THP-1/DBMSC+ H_2_O_2_ (THP-1 pretreated with DBMSCs and H_2_O_2_)] were harvested and then added to HUVEC at different THP-1:HUVEC ratios (2.5:1, 5:1, 10:1, and 20:1 THP-1:HUVEC) in the presence of 100 μM H_2_O_2_. Briefly, THP-1 were added to HUVEC that were initially seeded at a density of 5 × 10^3^ per well in 96-well tissue culture plates. After 24-h culture in a complete HUVEC culture medium at 37 °C in a cell culture incubator, HUVEC proliferation was examined using the MTS assay as previously described [12]. Before using DBMSCs and THP-1 in the proliferation assays, cells were treated with 25 μg/ml Mitomycin C to inhibit their proliferation as previously described [12]. Results were presented as means (± standard errors). Each experiment was performed in triplicate and repeated for five times with five independent preparations of DBMSCs and HUVEC. DBMSCs and THP-1 cultured alone were included as negative controls.

### Adhesion of monocyte to HUVEC

DBMSC effect on THP-1 adhesion to HUVECs was examined using our previously published method [12]. Briefly, H_2_O_2_-untreated THP-1 or pretreated with 100 μM H_2_O_2_ (TTHP-1) for 24 h were cocultured with H_2_O_2_-untreated DBMSCs (TTHP-1/UDBMSC) or with H_2_O_2_-treated DBMSCs (TTHP-1/TDBMSC) for 24 h at 5:1 THP-1:DBMSC ratio in a physical contact experiment by adding THP-1 to DBMSCs that were initially cultured on a plastic surface of 6-well culture plate for 24 h to allow cells to be fully adhered (Fig. 2). After 24-h incubation in a complete RPMI-1640 culture medium (above), THP-1 were harvested and then labelled with 5 μM green fluorescent cell tracker stain (5-chloromethylfluorescin diacetate; CMFDA; Molecular Probes, Life Technologies) for 4 h as previously described [12]. Following washing THP-1 with fresh RPMI-1640 culture medium, they were added to a monolayer layer of HUVEC at a ratio of 5THP-1:1HUVEC (HUVEC were initially cultured alone or with 100 μM H_2_O_2_ for 24 h). After incubation for 30 min, non-adherent THP-1 were gently removed by washing with PBS, and the fluorescence intensity of the THP-1 that had adhered to the monolayer of HUVEC was then measured at excitation 485 nm and emission 528 nm using a fluorescence microplate reader (Glomax Multi Detection System, Promega, Germany). Results were expressed as relative fluorescence intensity (RFI). Different ratios of HUVEC to THP-1 were evaluated. Experiments were performed in triplicate using HUVEC prepared from independent umbilical cord tissue and repeated three times.

### Measurement of glutathione reductase activity

HUVEC (HUVEC were initially co-cultured with DBMSCs and 100 μM H_2_O_2_ or cultured alone as described above) were washed twice with cold PBS, and they were then lysed as previously described [12, 13]. Total protein in the supernatant was then determined by Bradford method [12, 13].

The activity of glutathione reductase was measured using OxiSelect™ Glutathione Reductase Assay Kit (catalogue number STA-812, Cell Biolabs, San Diego, USA) as previously described by us [13]. This assay is based on the reduction of glutathione disulfide (oxidized glutathione) (GSSG) to reduced glutathione (GSH) by glutathione reductase, using NADPH as a donor for H. Subsequently, the chromogen reacts with the thiol group of GSH to produce a colored compound that absorbs at 405 nm. The glutathione reductase content in HUVEC samples is determined by comparison with the predetermined glutathione reductase standard curve. The assay was performed using 100-μl aliquots of HUVEC supernatant protein immediately after preparation (30 μg protein) added to phosphate buffer containing excess GSSG and NADPH. The level of change was determined at 405 nm using a standard curve performed. Three experiments were performed in triplicate using HUVEC and DBMSCs as indicated above.

### Measurement of thioredoxin reductase activity

Total protein was extracted from HUVEC (prepared as described above), and thioredoxin reductase (TrxR) activity (catalogue number 10007892, Cayman, Michigan, USA) was then evaluated as per the manufacturer’s instructions. This assay is based on the reduction of 5,5′-dithiobis (2-nitrobenzoic) acid (DTNB) with NADPH to 5-thio-2-nitrobenzoic acid, which generates a strong yellow color that can be measured at 412 nm. In the crude biological sample, glutathione reductase and glutathione peroxidase can also be reduced by DTNB. Therefore, TrxR specific inhibitor is used to determine the specific activity of TrxR. Therefore, the total DTNB reduction by the sample is initially estimated and the DTNB reduction by the sample in the presence of the TrxR specific inhibitor will then be estimated. The difference between the two results is the DTNB reduction due to TrxR activity. Three experiments were performed in triplicate using HUVEC and DBMSCs as indicated above.

### RNA isolation, cDNA synthesis, and real-time polymerase chain reaction (RT-PCR) analysis

The expression of 84 genes related to endothelial cell biology (catalogue number PAHS-015ZD-24, Qiagen, Hilden, Germany) by HUVEC was determined using QuantiTect Primer Assay (Qiagen, Hilden, Germany) in a real-time polymerase chain reaction (RT-PCR) as previously published [2]. Briefly, total RNA from HUVEC pretreated with DBMSCs and 100 μM H_2_O_2_ for 48 h was isolated, and cDNA was then synthesized using FastLane Cell cDNA kit and RT Primer Mix (Qiagen) as previously published [2]. After quantifying mRNA using QuantiTect SYBR Green PCR Kit (Qiagen), the real-time PCR reaction was performed in triplicate on the CFX96 real-time PCR detection system (BIO-RAD) as previously published [2]. To analyze the data, the CFX manager software (Bio-Rad, CA, USA) was used. The results were exported to Microsoft Excel for further analysis. The results were expressed as fold change by calculating the ΔΔ^−2^ values. The relative expression of an internal house-keeping gene as a loading control was used as provided in the kit. Experiments were performed in triplicate using HUVEC prepared from independent umbilical cord tissue and repeated three times.

### Flow cytometry

Cells were characterized by flow cytometry as previously described [12]. Briefly, cells (1 × 10^5^) were stained with monoclonal antibodies (Table 1) for 30 min. Cells were then washed twice by adding cold PBS and centrifuged at 150×*g* for 5 min at 8 °C. Unstained and isotype controls were used. Immunoreactivity to cell surface antibody markers or intracellular proteins was assayed by a BD FACS CANTO II (Becton Dickinson, NJ, USA) flow cytometer.

### Statistical analysis

Data were analyzed using the *t* test (unpaired *t* test, two tailed). These analyses were performed using GraphPad Prism 5. Results were considered to be statistically significant if *P* < 0.05.

## Results

### Isolation and characterization of DBMSCs

MSCs from *decidua basalis* of human term placenta were previously isolated and characterized by us [2]. DBMSCs at passage 3 were positive (> 95%) for MSC markers and negative for hematopoietic markers and were able to differentiate into adipocytes, chondrocytes, and osteocytes as previously report [2]. Subsequently, DBMSCs at passage 3 were used in all experiments.

### DBMSCs and H_2_O_2_ modulated the proliferation of HUVEC

To evaluate the effects of DBMSCs on endothelial cell functions, the proliferation of HUVEC cultured with DBMSCs in the presence or absence of 100 μM H_2_O_2_ was examined using the MTS assay. The viability HUVEC exposed to 100 μM H_2_O_2_ was more than 90% at all culture time points (24, 48, and 72 h). This was consistent with our previous report [13]. The exposure of HUVEC to concentrations higher than 100 μM H_2_O_2_ reduced their viability to less than 50%, as we previously reported [13]. Consequently, 100 μM H_2_O_2_ was used in this study.

**Fig. 3:**
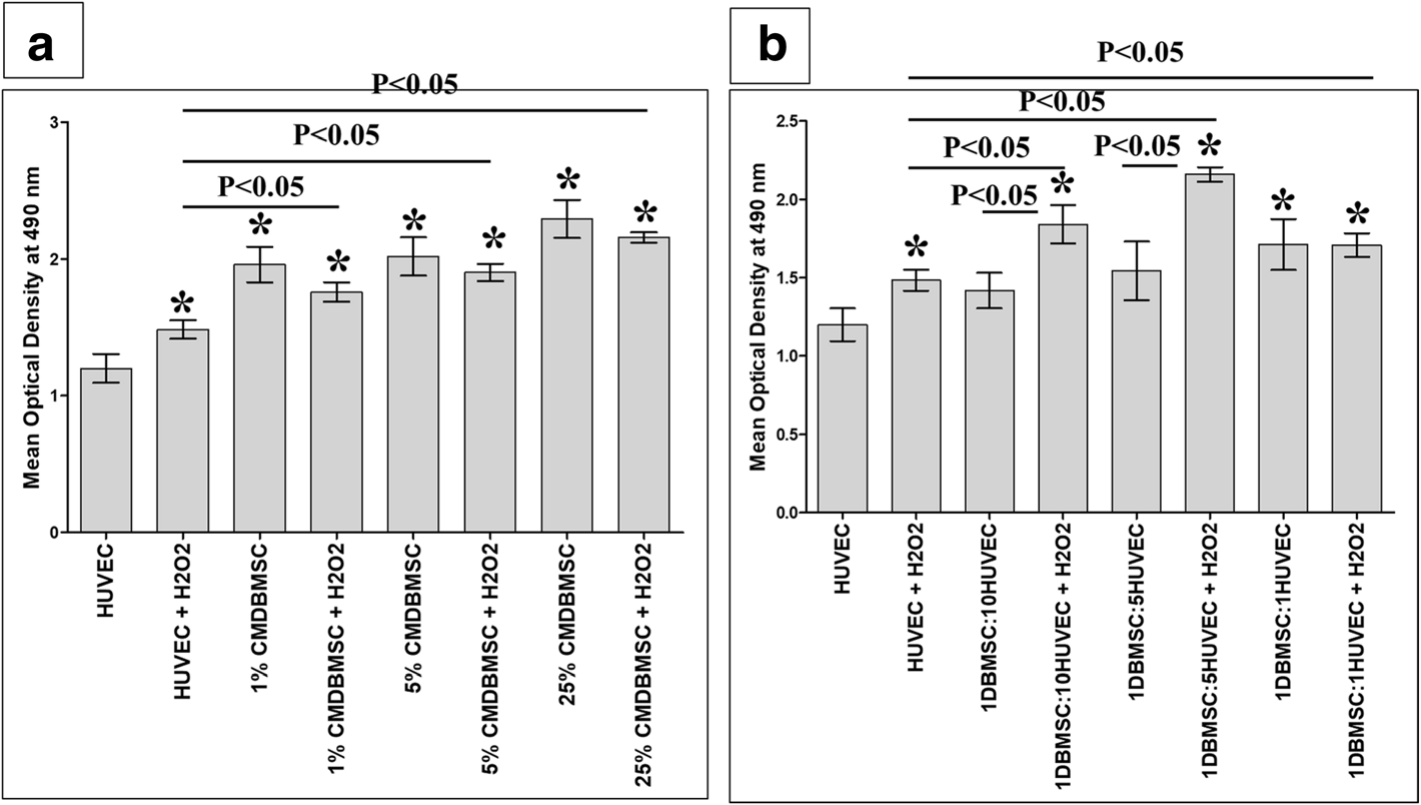
Proliferation of HUVEC measured by MTS. As compared to untreated HUVEC, the proliferation of HUVEC significantly increased in response to H_2_O_2_ alone or with different concentrations (1%, 5%, and 25%) of CMDBMSC in the presence of 100 μM H_2_O_2_ (**a**) and with different ratios of DBMSC to HUVEC (1:1, 1:5, and 1:10) in the presence of 100 μM H_2_O_2_ (**b**). As compared to HUVEC treated with H_2_O_2_ (HUVEC + H_2_O_2_), the proliferation of HUVEC significantly increased in response to different concentrations (1%, 5% and 25%) of CMDBMSC in presence of 100 μM H_2_O_2_ (**a**) and with different ratios of DBMSC to HUVEC (1:1, 1:5, and 1:10) in presence of 100 μM H_2_O_2_ (**b**). As compared to untreated HUVEC, HUVEC proliferation significantly increased in response to different concentrations (1%, 5%, and 25%) of CMDBMSC (**a**), and at a high ratio of DBMSC to HUVEC (1:1) (**b**). As compared to HUVEC cultured with CMDBMSC, HUVEC proliferation did not significantly change in response to H_2_O_2_ and CMDBMSCs, *P* > 0.05 (**a**). As compared to HUVEC cultured with low ratios of DBMSCs to HUVEC (1:5 and 1:10), HUVEC proliferation significantly increased in response to H_2_O_2_ and DBMSCs (**b**). *P < 0.05. Bars represent standard errors. Each experiment was performed in triplicate and repeated for five times with five independent preparations of DBMSCs and HUVEC

The effects of H_2_O_2_ on HUVEC proliferation maintained throughout the culture times (24, 48, and 72 h), but the addition of DBMSCs (CMDBMSC and DBMSCs) significantly (*P* < 0.05) induced the effect of H_2_O_2_ on HUVEC proliferation after 48 h in culture at all examined concentrations of CMDBMSC and ratios of DBMSCs, respectively, as compared to untreated HUVEC or H_2_O_2_-treated HUVEC, *P* < 0.05 (Fig. 3a, b), and had no significant changes on the effect of H_2_O_2_ on HUVEC proliferation after 24 and 72 h in culture, *P* > 0.05. Consequently, the culture time used in this study was 48 h. HUVEC proliferation was also significantly increased in response to at all examined concentrations of CMDBMSC, *P* < 0.05 (Fig. 3a), and only at a high ratio of DBMSCs to HUVEC (1:1), *P* < 0.05 (Fig. 3b) as compared to untreated HUVEC. As compared to HUVEC cultured with CMDBMSC, HUVEC proliferation did not significantly change in response to H_2_O_2_ and CMDBMSCs, *P* > 0.05 (Fig. 3a). As compared to HUVEC cultured with low ratios of DBMSCs to HUVEC (1:5 and 1:10), HUVEC proliferation significantly increased in response to H_2_O_2_ and DBMSCs, *P* < 0.05 (Fig. 3b).

### The effects of DBMSCs and H_2_O_2_ on HUVEC proliferation are reversible

**Fig. 4:**
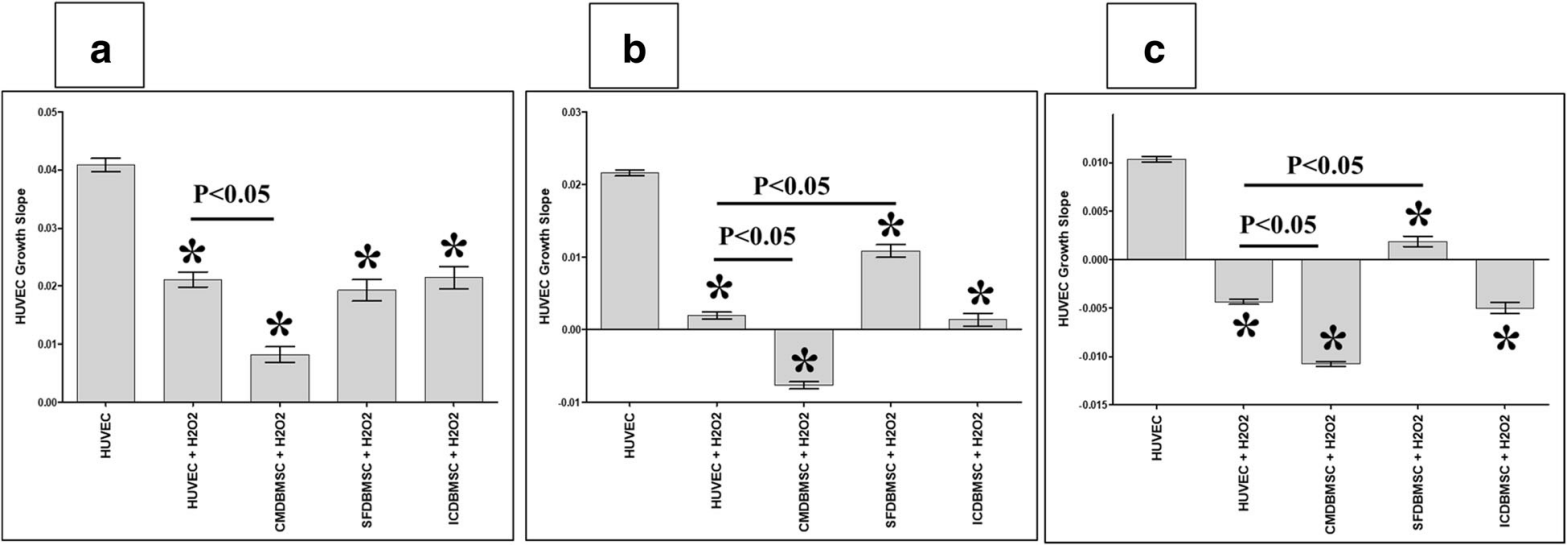
The proliferation of HUVEC after removing the effects of DBMSCs and H_2_O_2_. HUVEC were initially cultured with DBMSCs and 100 μM H_2_O_2_ for 48 h and then used in a proliferation assay using the xCELLigence real-time cell analyzer. After 24 (**a**), 48 (**b**), and 72 (**c**) hours, the proliferation of HUVEC pretreated with H_2_O_2_ (HUVEC + H_2_O_2_) or with H_2_O_2_ and CMDBMSC (CMDBMSC + H_2_O_2_) or with H_2_O_2_ and SFDBMSC (SFDBMSC + H_2_O_2_) or with H_2_O_2_ and ICDBMSC (ICDBMSC + H_2_O_2_) significantly reduced as compared to untreated HUVEC. After 24, 48, and 72 h, the proliferation of HUVEC pretreated with H_2_O_2_ and CMDBMSC (CMDBMSC + H_2_O_2_) significantly reduced as compared to HUVEC pretreated with H_2_O_2_ (HUVEC + H_2_O_2_) (**a**–**c**). The proliferation of HUVEC pretreated with H_2_O_2_ and SFDBMSC (SFDBMSC + H_2_O_2_) significantly increased after 48 and 72 h as compared to H_2_O_2_-treated HUVEC (HUVEC + H_2_O_2_) (**b**, **c**), but did not change significantly after 24 h (*P* > 0.05) (**a**). The proliferation of HUVEC pretreated with H_2_O_2_ and ICDBMSC (ICDBMSC + H_2_O_2_) was not significantly changed (*P* > 0.05) as compared to H_2_O_2_-treated HUVEC (HUVEC + H_2_O_2_) (**a**–**c**). Each experiment was performed in triplicate and repeated with five independent HUVEC (passages 3–5) and DBMSC (passage 3) preparations. *P < 0.05. Bars represent standard errors

To evaluate the reversibility of DBMSC effects on the proliferation of H_2_O_2_-treated HUVEC, HUVEC were initially cultured with different treatments of DBMSCs in the presence of 100 μM H_2_O_2_ for 48 h and their proliferation was then determined using the xCELLigence system. After 24, 48, and 72 h, the proliferation of HUVEC pretreated with H_2_O_2_ (HUVEC + H_2_O_2_) and HUVEC pretreated with H_2_O_2_ and with different treatments of DBMSCs (CMDBMSC + H_2_O_2_, SFDBMSC + H_2_O_2_, and ICDBMSC + H_2_O_2_) significantly reduced as compared to untreated HUVEC, *P* < 0.05 (Fig. 4a–c). Similarly, after 24, 48, and 72 h, the proliferation of HUVEC pretreated with H_2_O_2_ and CMDBMSC (CMDBMSC + H_2_O_2_) significantly reduced as compared to HUVEC pretreated with H_2_O_2_, *P* < 0.05 (Fig. 4a–c). In contrast, after 48 and 72 h, and as compared to HUVEC pretreated with H_2_O_2_, the proliferation of HUVEC pretreated with H_2_O_2_ and SFDBMSC (SFDBMSC + H_2_O_2_) significantly increased (*P* < 0.05), but did not change significantly after 24 h, *P* > 0.05 (Fig. 4a–c). In addition, the culture with ICDBMSC (ICDBMSC + H_2_O_2_) did not significantly affect the proliferation of HUVEC pretreated with H_2_O_2_ as compared to HUVEC pretreated with H_2_O_2_ at all examined time points in culture, *P* > 0.05 (Fig. 4a–c).

### DBMSCs and H_2_O_2_ modulated HUVEC adhesion

**Fig. 5:**
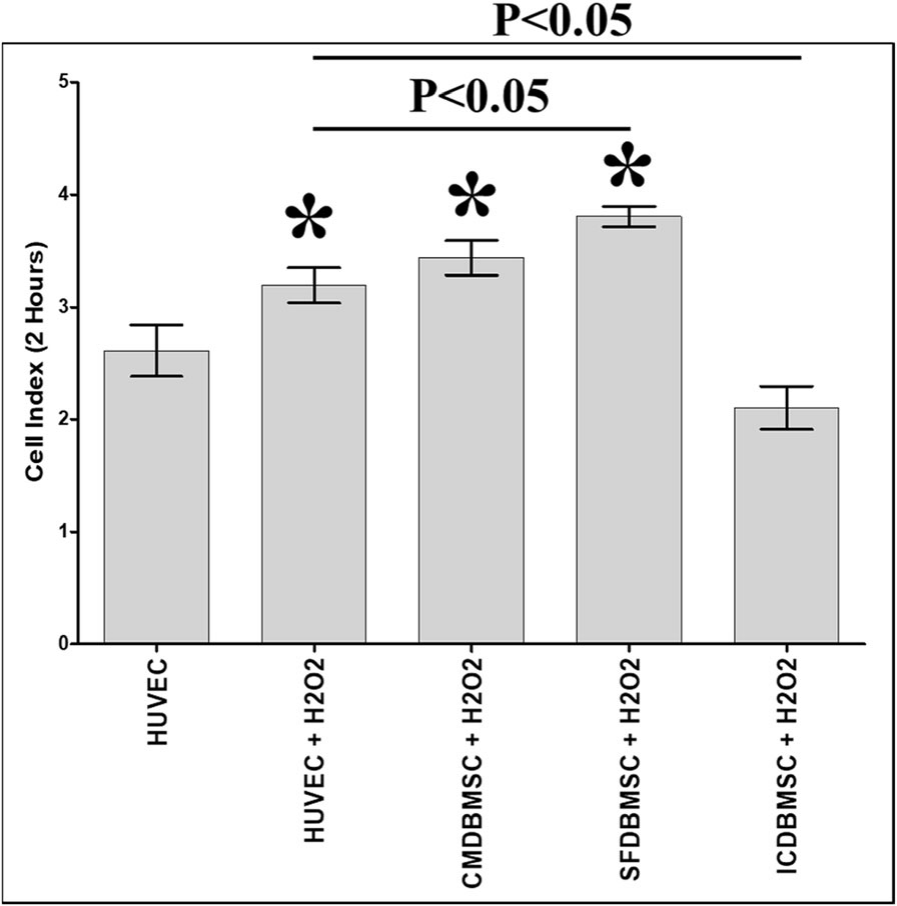
The adhesion of HUVEC after removing the effects of DBMSCs and H_2_O_2_. HUVEC were initially cultured with DBMSC with 100 μM H_2_O_2_ for 48 h and then used in an adhesion assay using the xCELLigence real-time cell analyzer. After 2 h, the adhesion of HUVEC pretreated with H_2_O_2_ alone (HUVEC + H_2_O_2_) or with H_2_O_2_ and CMDBMSC (CMDBMSC + H_2_O_2_) or with H_2_O_2_ and SFDBMSC (SFDBMSC + H_2_O_2_) significantly increased as compared to untreated HUVEC while the adhesion of HUVEC pretreated with H_2_O_2_ and ICDBMSC (ICDBMSC + H_2_O_2_) reduced, but not significantly (*P* > 0.05). After 2 h and as compared with HUVEC pretreated with H_2_O_2_ (HUVEC + H_2_O_2_), the adhesion of HUVEC pretreated with H_2_O_2_ and SFDBMSC (SFDBMSC + H_2_O_2_) or ICDBMSC (ICDBMSC + H_2_O_2_) significantly increased and decreased, respectively. Each experiment was performed in triplicate and repeated with five independent HUVEC (passages 3–5) and DBMSC (passage 3) preparations. *P < 0.05. Bars represent standard errors

To study the effects of DBMSCs on the adhesion of H_2_O_2_-treated HUVEC, HUVEC were initially cultured with different treatments of DBMSCs in the presence of 100 μM H_2_O_2_ for 48 h and their adhesion was then determined using the xCELLigence system. After 2 h, the adhesion of HUVEC pretreated with H_2_O_2_ (HUVEC + H_2_O_2_) and HUVEC pretreated with H_2_O_2_ and different treatments of DBMSCs (CMDBMSC + H_2_O_2_ and SFDBMSC+ H_2_O_2_) significantly increased as compared to untreated HUVEC, *P* < 0.05 (Fig. 5). In contrast, culturing with ICDBMSC (ICDBMSC + H_2_O_2_) decreased the adhesion of H_2_O_2_-treated HUVEC, but not significantly as compared to untreated HUVEC, *P* > 0.05 (Fig. 5). As compared to H_2_O_2_-treated HUVEC, the adhesion of H_2_O_2_-treated HUVEC cultured with SFDBMSC and ICDBMSC (SFDBMSC+ H_2_O_2_ and ICDBMSC+ H_2_O_2_) significantly increased and reduced, respectively (*P* < 0.05) (Fig. 5). In contrast, culturing with CMDBMSC (CMDBMSC + H_2_O_2_) had no significant changes on the adhesion of H_2_O_2_-treated HUVEC as compared to H_2_O_2_-treated HUVEC cultured alone, *P* > 0.05 (Fig. 5).

### DBMSCs and H_2_O_2_ modulated HUVEC migration

**Fig. 6:**
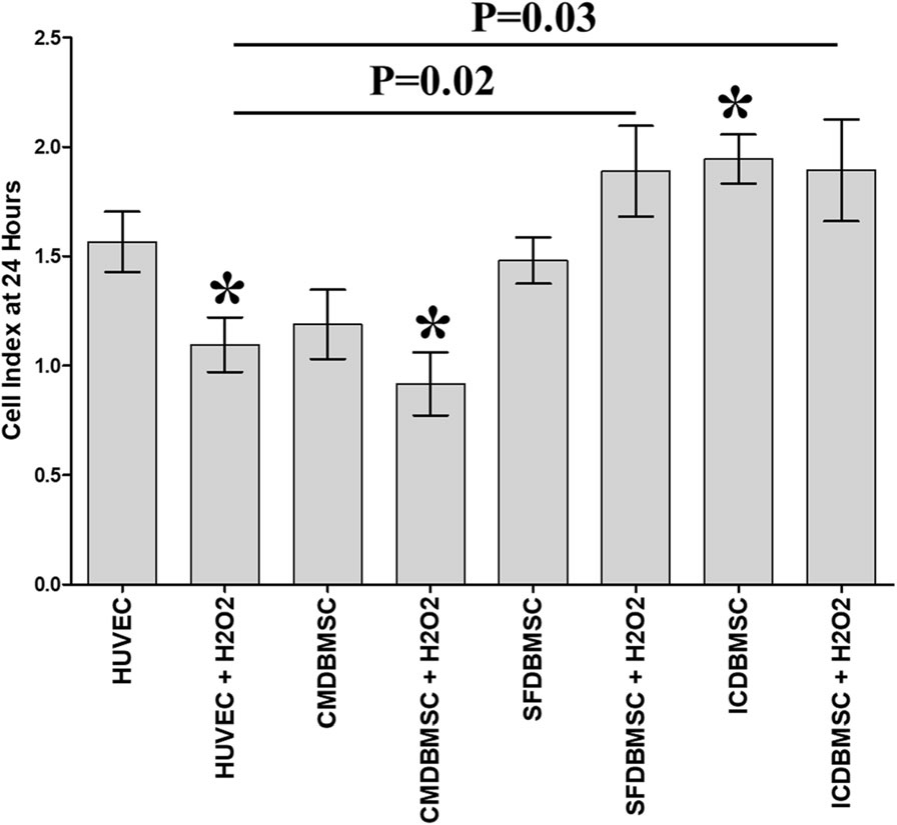
The migration of HUVEC after removing the effects of DBMSCs and H_2_O_2_. HUVEC were initially cultured with DBMSC with 100 μM H_2_O_2_ for 48 h and then used in a migration assay using the xCELLigence real-time cell analyzer. After 24 h, the migration of HUVEC pretreated with H_2_O_2_ alone (HUVEC + H_2_O_2_) or with H_2_O_2_ and CMDBMSC (CMDBMSC + H_2_O_2_) significantly reduced as compared to untreated HUVEC while the migration of HUVEC pretreated with H_2_O_2_ and with SFDBMSC (SFDBMSC + H_2_O_2_) or H_2_O_2_ and ICDBMSC (ICDBMSC + H_2_O_2_) increased, but not significantly (*P* > 0.05). After 24 h, the migration of HUVEC pretreated with H_2_O_2_ and with SFDBMSC (SFDBMSC + H_2_O_2_) or with ICDBMSC (ICDBMSC + H_2_O_2_) significantly increased as compared to HUVEC pretreated with H_2_O_2_ (HUVEC + H_2_O_2_). After 24 h and as compared to untreated HUVEC, the migration of HUVEC treated with CMDBMSC or SFDBMSC did not significantly change, *P* > 0.05. In contrast, the migration of HUVEC treated with ICDBMSC significantly increased as compared to untreated HUVEC after 24 h. After 24 h and as compared to HUVEC treated with CMDBMSC, SFDBMSC, or ICDBMSC, the migration of HUVEC treated with H_2_O_2_ in the presence of CMDBMSC or SFDBMSC or ICDBMSC did not significantly change, *P* > 0.05. Each experiment was performed in triplicate and repeated with five independent HUVEC (passages 3–5) and DBMSC (passage 3) preparations. *P < 0.05. Bars represent standard errors

To further study the effect of DBMSCs and H_2_O_2_ on the migration of endothelial cells, HUVEC were initially cultured with different treatment of DBMSCs in the presence of 100 μM H_2_O_2_ (CMDBMSC + H_2_O_2_, SFDBMSC + H_2_O_2_, and ICDBMSC + H_2_O_2_) or in the absence of H_2_O_2_ (CMDBMSC, SFDBMSC, and ICDBMSC) for 48 h and then re-cultured in a 16-well migration culture plate and monitored using the xCELLigence system. After 24 h, the migration of H_2_O_2_-treated HUVEC (HUVEC + H_2_O_2_) cultured alone or with CMDBMSC (ICDBMSC + H_2_O_2_) significantly reduced as compared to untreated HUVEC, *P* < 0.05 (Fig. 6). In contrast, the migration of H_2_O_2_-treated HUVEC cultured with SFDBMSC and ICDBMSC (SFDBMSC + H_2_O_2_ and ICDBMSC + H_2_O_2_) did not change significantly as compared to untreated HUVEC (*P* > 0.05), but significantly increased as compared to H_2_O_2_-treated HUVEC, *P* < 0.05 (Fig. 6). The incubation with CMDBMSC (CMDBMSC+ H_2_O_2_) reduced the migration of H_2_O_2_-treated HUVEC, but not significantly (*P* > 0.05) as compared to H_2_O_2_-treated HUVEC (Fig. 6). After 24 h, the migration of HUVEC treated with CMDBMSC or SFDBMSC did not significantly change as compared to untreated HUVEC, *P* > 0.05 (Fig. 6). In contrast, the migration of HUVEC treated with ICDBMSC significantly increased as compared to untreated HUVEC, *P* < 0.05 (Fig. 6). As compared to HUVEC treated with CMDBMSC, SFDBMSC, or ICDBMSC, the migration of HUVEC treated with H_2_O_2_ in the presence of CMDBMSC or SFDBMSC or ICDBMSC did not significantly change, *P* > 0.05 (Fig. 6).

### DBMSCs reduced the stimulatory effect of monocytes and H_2_O_2_ on HUVEC proliferation

**Fig. 7:**
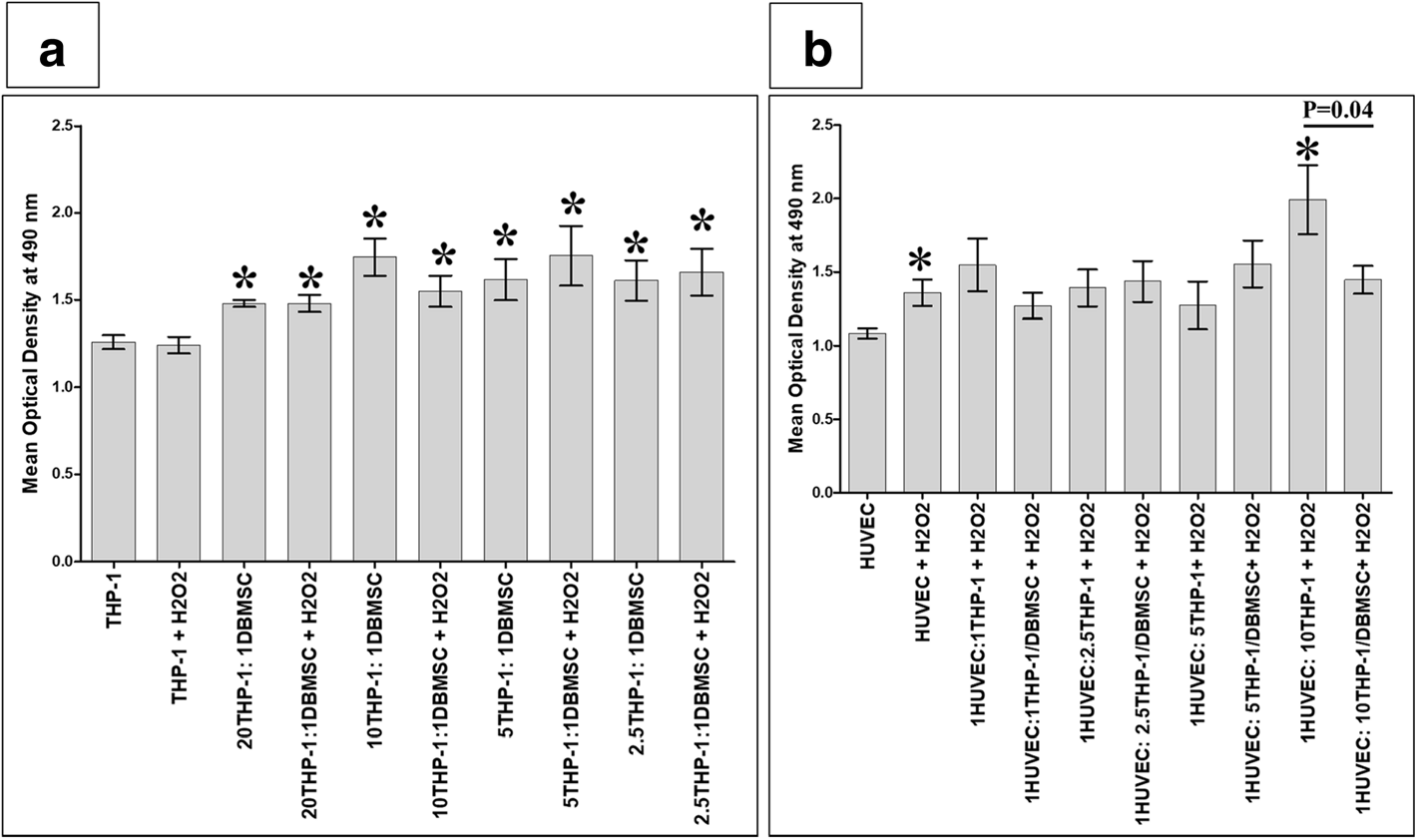
The proliferation of monocytes (THP-1) and HUVEC evaluated by the MTS assay. After 24 h and as compared to untreated THP-1, the proliferation of THP-1 in the presence of H_2_O_2_ and DBMSCs (THP-1/DBMSC + H_2_O_2_) significantly increased at different THP-1 and DBMSC ratios (20:1, 10:1, 5:1, and 2.5:1) (**a**). After 24 h and as compared with untreated HUVEC, the proliferation of H_2_O_2_-treated HUVEC (HUVEC + H_2_O_2_) and H_2_O_2_-treated HUVEC in the presence of THP-1 pretreated with H_2_O_2_ (THP-1 + H_2_O_2_) at 1:10 HUVEC:THP-1 ratio significantly increased (**b**). The proliferation of H_2_O_2_-treated HUVEC in the presence of THP-1 pretreated with H_2_O_2_ and DBMSCs (THP-1/DBMSC + H_2_O_2_) significantly reduced as compared with H_2_O_2_-treated HUVEC cultured with THP-1 pretreated with H_2_O_2_ (**b**). After 24 h culture with DBMSCs and as compared to monocytes cultured alone, the proliferation of THP-1 significantly increased at all examined ratios of monocytes and DBMSCs (**a**). As compared to monocytes cultured with H_2_O_2_ alone, the proliferation of monocyte cultured with DBMSCs did not significantly change, *P* > 0.05 (**a**). Each experiment was performed in triplicate and repeated for five times with five independent preparations of DBMSCs and HUVEC. *P < 0.05. Bars represent standard errors

To study the effect of DBMSCs on the interaction between monocytes and H_2_O_2_ (THP-1 + H_2_O_2_) on the proliferation of HUVEC, the effect of DBMSCs on the proliferation of monocytes in the presence of H_2_O_2_ (THP-1:DBMSC + H_2_O_2_) was first examined using the MTS assay. Then, the proliferation of HUVEC in response to monocytes pre-cultured with DBMSCs and H_2_O_2_ (HUVEC:THP-1/DBMSC + H_2_O_2_) was also evaluated. After 24-h culture with DBMSCs, the proliferation of monocytes cultured with H_2_O_2_ and DBMSCs (THP-1:DBMSC + H_2_O_2_) at all examined ratios of monocytes and DBMSCs significantly increased, *P* < 0.05 (Fig. 7a) as compared to monocytes cultured alone or with H_2_O_2_ (THP-1 + H_2_O_2_). This stimulatory effect of DBMSCs on the proliferation of monocytes is reversible and time dependent (data not shown). The effect of DBMSCs on the proliferation of monocytes (THP-1) alone was also evaluated. After 24-h culture with DBMSCs, THP-1 proliferation at all examined ratios of monocytes and DBMSCs significantly increased, *P* < 0.05 (Fig. 7a) as compared to monocytes cultured alone. Following the addition of H_2_O_2_ to monocyte cultured with DBMSCs, monocyte proliferation did not significantly change as compared to monocytes cultured with H_2_O_2_ alone, *P* > 0.05 (Fig. 7a).

After 24 h, the proliferation of HUVEC cultured in H_2_O_2_ significantly increased after the addition of a high ratio of monocytes pretreated with H_2_O_2_ (THP-1 + H_2_O_2_) to HUVEC (1HUVEC:10THP-1 + H_2_O_2_), and this stimulatory effect of monocytes pretreated with H_2_O_2_ on HUVEC proliferation was significantly reduced (*P* < 0.05) by monocytes pretreated with DBMSCs and H_2_O_2_ (THP-1/DBMSC + H_2_O_2_), *P* < 0.05 (Fig. 7b). This stimulatory effect of monocytes pretreated with H_2_O_2_ (THP-1 + H_2_O_2_) on the proliferation of HUVEC cultured in H_2_O_2_ and the inhibitory effect of DBMSCs on monocytes pretreated with H_2_O_2_ (THP-1/DBMSC + H_2_O_2_) inducing the proliferation of HUVEC cultured in H_2_O_2_ are reversible and time dependent (data not shown). The effects of DBMSCs on the proliferative responses of HUVEC cultured with or without H_2_O_2_ and monocytes pretreated with or without H_2_O_2_ were not significantly changed, *P* > 0.05 (data not shown).

### DBMSCs reduced the adhesion of H_2_O_2_-treated monocytes to HUVEC

**Fig. 8:**
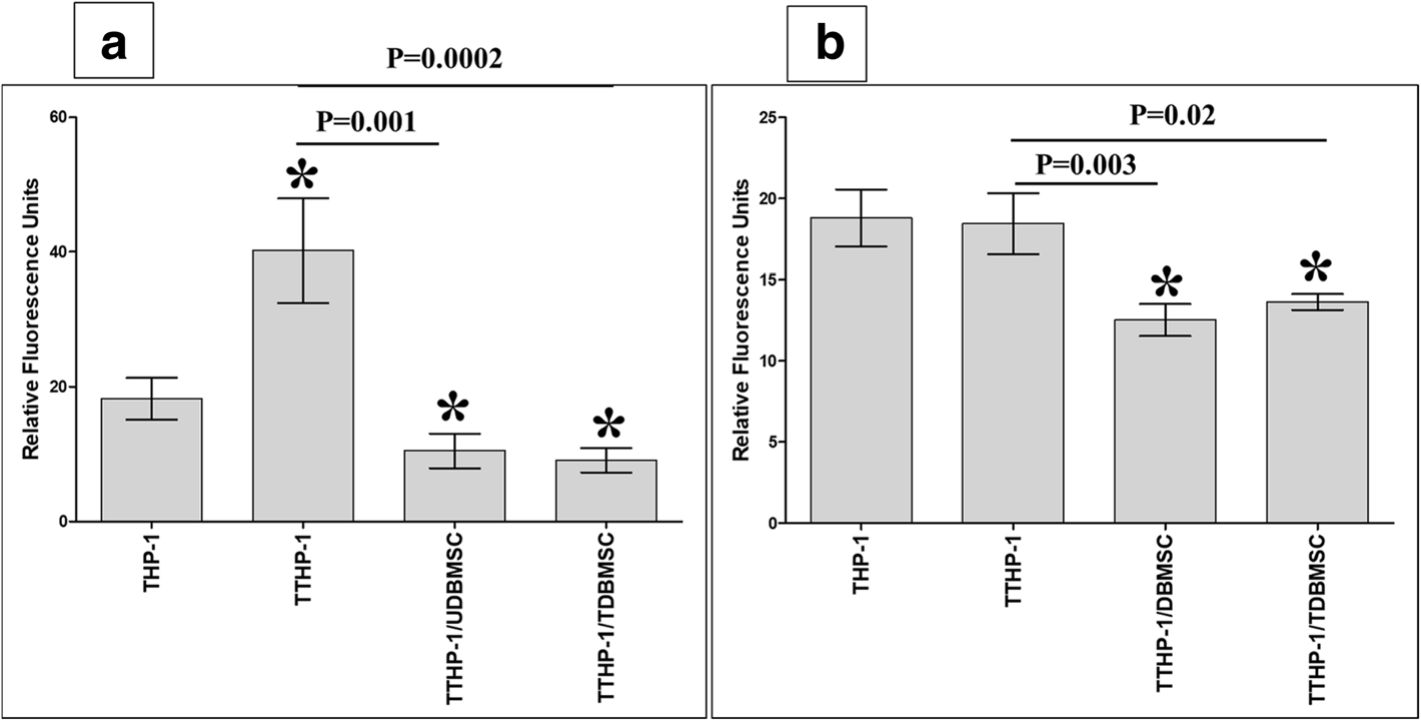
The adhesion of monocytes (THP-1) to HUVEC was evaluated by measuring THP-1 fluorescence intensity using a fluorescence microplate reader. THP-1 were initially cultured alone (THP-1) or with 100 μM H_2_O_2_ (TTHP-1) for 24 h and then cultured with DBMSCs (5:1 THP-1:DBMSC ratio) that were initially cultured alone (UDBMSC) or with 100 μM H_2_O_2_ (TDBMSC) for 24 h. After 24 h culture, THP-1 were labelled with 5 μM green fluorescent cell tracker stain CMFDA and added to HUVEC monolayer (HUVEC were initially cultured with or without 100 μM H_2_O_2_ for 24 h). As compared to untreated THP-1, the adhesion of TTHP-1 to H_2_O_2_ untreated HUVEC significantly increased while the adhesion of TTHP-1/UDBMSCs and TTHP-1/TDBMSC to H_2_O_2_ untreated HUVEC significantly reduced after 30 min (**a**). As compared to TTHP-1, the adhesion of TTHP-1/UDBMSCs and TTHP-1/TDBMSC to H_2_O_2_ untreated HUVEC significantly reduced after 30 min (**a**). As compared to untreated THP-1, the adhesion of TTHP-1 to H_2_O_2_ pretreated HUVEC was not significantly changed (*P* > 0.05) after 30 min while the adhesion of TTHP-1/UDBMSCs and TTHP-1/TDBMSC to H_2_O_2_ pretreated HUVEC significantly reduced after 30 min as compared to untreated THP-1 and TTHP-1 (**b**). Each experiment was performed in triplicate and repeated for five times with five independent preparations of DBMSCs and HUVEC. *P < 0.05. Bars represent standard errors

To evaluate the effect of DBMSCs on the adhesion of monocytes to endothelial cells, monocytes pretreated with H_2_O_2_ (TTHP-1) were cultured with H_2_O_2_-untreated DBMSCs (UDBMSC) or DBMSC pretreated with H_2_O_2_ (TDBMSC) for 24 h, and THP-1 were then harvested and labelled with the green fluorescent dye CMFDA and added to endothelial cells (HUVEC were initially cultured alone or with 100 μM H_2_O_2_ for 24 h) in an adhesion assay. Results showed that the adhesion of monocytes pretreated with H_2_O_2_ to endothelial cells (HUVEC were initially cultured alone or with H_2_O_2_) was significantly reduced after culturing with UDBMSC or TDBMSC, *P* < 0.05 (Fig. 8a, b). There was no difference in the adhesion of H_2_O_2_-untreated monocytes and H_2_O_2_-treated monocytes to endothelial cells by DBMSCs (data not shown).

**Fig. 9:**
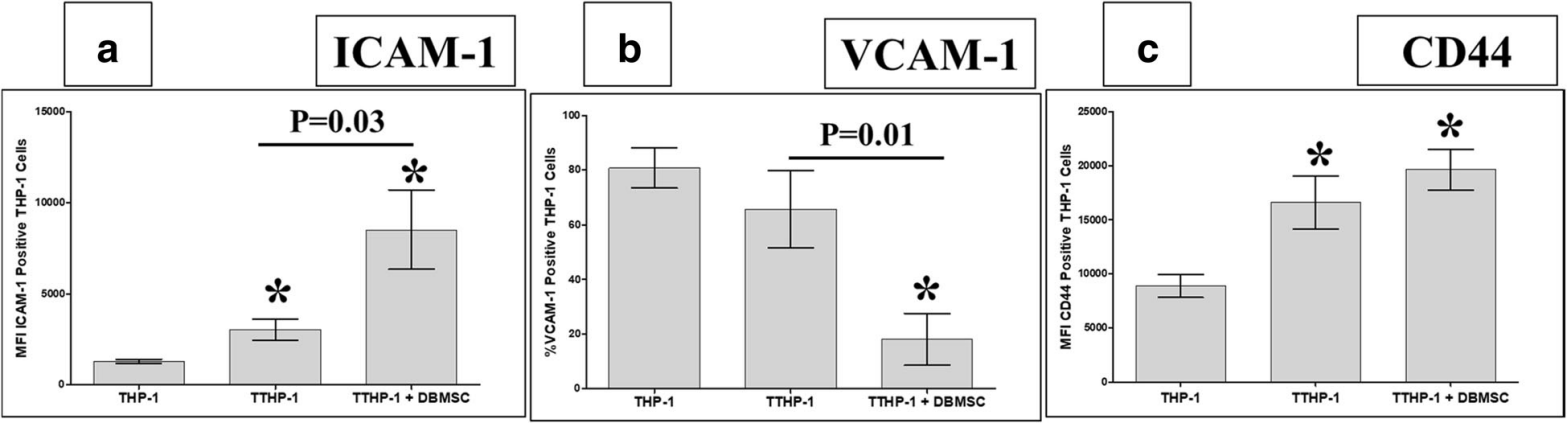
The flow cytometric analysis of monocytes (THP-1) expression of ICAM-1, VCAM-1, and CD44. THP-1 were initially cultured with 100 μM H_2_O_2_ (TTHP-1) for 24 h and then cultured with DBMSCs at 5:1 THP-1:DBMSC ratio (TTHP-1/DBMSC). The flow cytometry was then evaluated after 24 h culture. The expression of ICAM-1 by TTHP-1 and by TTHP-1/DBMSC significantly increased as compared with H_2_O_2_-untreated THP-1 (**a**). As compared to TTHP-1, the expression of ICAM-1 by TTHP-1/DBMSC significantly increased (**a**). As compared with untreated THP-1, TTHP-1 expression of VCAM-1 was not significantly changed (*P* > 0.05) while significantly increased by TTHP-1/DBMSC (**b**). As compared with TTHP-1, TTHP-1/ DBMSC expression of VCAM-1 significantly increased (**b**). TTHP-1 and TTHP-1/DBMSC expression of CD44 significantly increased as compared to untreated THP-1 while TTHP-1/DBMSC expression of CD44 was not significantly changed as compared with TTHP-1 (**c**). Each experiment was performed in triplicate and repeated for five times with five independent preparations of DBMSCs and HUVEC. *P < 0.05. Bars represent standard errors

Next, we studied that the inhibitory effects of DBMSCs on the adhesion of monocytes pretreated with H_2_O_2_ to endothelial cells were mediated by modulating the expression of adhesion molecules by monocytes pretreated with H_2_O_2_. A range of adhesion molecules were studied by flow cytometry and expression recorded as median fluorescence intensity or as a percentage of cells. After 24 h, the expression of ICAM-1 in monocytes pretreated with H_2_O_2_ (TTHP-1) was significantly increased as compared to H_2_O_2_-untreated monocytes (THP-1), and culturing with DBMSCs (TTHP-1 + DBMSC) significantly increased ICAM-1 expression in TTHP-1, *P* < 0.05 (Fig. 9a). Similarly, the expression of CD44 in TTHP-1 and TTHP-1 + DBMSC was significantly increased as compared to THP-1 (*P* < 0.05), and there was no difference in CD44 expression between TTHP-1 and TTHP-1 + DBMSC (Fig. 9c). In contrast, the expression of VCAM-1 in TTHP-1was not significantly changed as compared to THP-1 (*P* < 0.05), but the culture with DBMSCs (TTHP-1 + DBMSC) significantly decreased VECAM-1 expression in TTHP-1 (Fig. 9b).

### DBMSCs increased the activities of glutathione and thioredoxin reductases in H_2_O_2_-treated HUVEC

Cells are protected from injury induced by oxidative stress by employing several antioxidant defense mechanisms [14]. To address the possibility that DBMSCs can protect endothelial cells from injury induced by H_2_O_2_, we examined the effect of DBMSCs on the activities of glutathione and thioredoxin reductases (an antioxidant enzyme) in H_2_O_2_-treated endothelial cells.

Glutathione reductase activity in H_2_O_2_-treated HUVEC cultured with CMDMSC, SFDBMSC, and ICDBMSC is significantly higher than H_2_O_2_-treated HUVEC (*P* < 0.05). At baseline, the level of glutathione reductase in HUVEC was 77.5 mU/mL ± 4.87 mU/mL. Exposure of HUVEC to 100 μM H_2_O_2_ for 48 h, the level of glutathione reductase was 29.50 ± 2.55. The level of glutathione reductase levels in H_2_O_2_-treated endothelial cell cultured with CMDBMSC, SFDBMSC, and ICDBMSC were 56.90 mU/mL ± 3.11 mU/mL, 62.38 mU/mL ± 3.01 mU/mL, and 65.49 mU/mL ± 3.95 mU/mL, respectively. As compared to H_2_O_2_-treated HUVEC, there were an approximately 1.92-, 2.11-, and 2.22-fold increase in the levels of glutathione reductase in H_2_O_2_-treated HUVEC cultured with CMDMSC, SFDBMSC, and ICDBMSC, respectively, *P* < 0.05.

Thioredoxin reductase activity in H_2_O_2_-treated HUVEC cultured with CMDMSC, SFDBMSC, and ICDBMSC is significantly higher than H_2_O_2_-treated HUVEC (*P* < 0.05). At baseline, the activity of thioredoxin reductase was 91.67 ± 10.14 mU/10^6^ cell. Exposure of HUVEC to 100 μM H_2_O_2_ for 48 h, the activity of the enzyme was reduced to 43.33 ± 10.73 mU/10^6^ cell. The activity of thioredoxin reductase in H_2_O_2_-treated endothelial cell cultured with CMDBMSC, SFDBMSC, and ICDBMSC was 79.33 ± 7.21 mU/10^6^ cell, 88.33 ± 9.28 mU/10^6^ cell, and 83.33 ± 9.28 mU/10^6^ cell, respectively. As compared to H_2_O_2_-treated HUVEC, there were an approximately 1.83-, 2.03-, and 1.92-fold increase in the activity of thioredoxin reductase in H_2_O_2_-treated HUVEC cultured with CMDBMSC, SFDBMSC, and ICDBMSC, respectively. These data suggest that culturing H_2_O_2_-treated HUVEC with DBMSCs can protect endothelial cells from oxidative stress induced by H_2_O_2_.

### DBMSCs modulated the effect of H_2_O_2_ on the expression of genes important in endothelial cell functions

The expression of genes mediating endothelial cell functions was studied after culturing endothelial cells with H_2_O_2_ in the presence or absence of DBMSCs for 48 h and then analyzed and assessed using the real-time PCR assay. Results show that DBMSCs modulated H_2_O_2_ effects on endothelial cell expression of genes underlying many of endothelial cell functional activities including survival, apoptosis, injury, fibrosis formation, inflammation, angiogenesis, permeability, thrombus formation, and leukocyte adhesion as well as infiltration as compared to untreated endothelial cells (Tables 2, 3, 4, 5, and 6).

**Table 2:** DBMSCs modulate the expression of genes involved in endothelial cell (EC) survival, apoptosis, injury, fibrosis formation, and inflammation. THUVEC (HUVEC were cultured with 100 μM H2O2 for 48 h). TDBMSC (HUVEC were cultured with DBMSC and 100 μM H_2_O_2_ for 48 h)

#	Gene symbol	Gene full name	THUVEC mean ΔΔ^−2^ values	TDBMSC Mean ΔΔ^−2^ values	Fold change (TDBMSC Vs. THUVEC) *P* < 0.05	Biological activities
1	BCL2	B-cell Lymphoma 2	2	23	11.5-fold ↑	Induce EC survival
2	EDN1	Endothelin-1	12	82	> 6.64-fold ↑	
3	EDNRA	Endothelin-1 (ET-1) Receptor A	3	9	3-fold ↑	
4	HMOX1	Heme Oxygenase-1	7	158	20-fold ↑	
5	KDR	Vascular Endothelial Growth Factor Receptor 3 (VEGFR3)	2	52	22.57-fold ↑	
6	MMP2	Matrix Metallopeptidase 2	9	36	4-fold ↑	
7	SPHK1	Sphingosine Kinase 1	44	840,958	19112.68-fold ↑	
8	MMP9	Matrix Metallopeptidase 9	1	6	6-fold ↑	
9	PECAM1	Platelet endothelial cell adhesion molecule	1090	7242	6.64-fold ↑	
10	TNFSF10	TNF-related apoptosis-inducing ligand (TRAIL)	7	30	> 4.20-fold ↑	
11	TYMP	Thymidine Phosphorylase	5	27	> 5-fold ↑	
12	FAS	Fas cell surface death receptor	3	0.76	> 3-fold ↓	Induce EC apoptosis
13	PF4	Platelet Factor 4	21	0.69	> 30-fold ↓	
14	HIF1α	Hypoxia-Inducible Factor-1α	159	11	> 14-fold ↓	Induce EC injury
15	SERPINE1 (PAI-1)	Type 1 plasminogen activator inhibitor	997	248	4-fold ↓	
16	TIMP1	TIMP Metallopeptidase Inhibitor 1	3	1	3-fold ↓	
17	ACE	Angiotensin I Converting Enzyme	15,765	5	3153-fold ↓	
18	F2R	Coagulation Factor II Thrombin Receptor	20	1	20-fold ↓	Induce EC inflammation
19	ADAM17	A disintegrin and Metalloprotease 17	256	4	64-fold ↓	
20	FLT1	Vascular Endothelial Growth Factor Receptor 1	23	12	1.91-fold ↓	
21	TNF-α	Tumor Necrosis Factor-α	591	2	> 295-fold ↓	
22	PTK2	PTK2 protein tyrosine kinase 2 (PTK2) or focal adhesion kinase (FAK),	8	2	4-fold ↓	Inhibits inflammation and fibrosis
23	PLG	Plasminogen	8	90	> 11-fold ↑	Inhibits inflammation and, promotes fibrin clearance
24	F3	Coagulation Factor III, Tissue Factor	30	0.06	500-fold ↓	Induces EC injury by induction of fibrin and thrombus formation
25	THBD	Thrombomodulin	1013	1	1013-fold ↓	
26	THBS1	Thrombospondin 1 (TSP-1)	1097	3	> 365-fold ↓	Induces EC inflammation and injury
27	TFPI	Tissue Factor Pathway Inhibitor	11	23	~ 2-fold ↑	Inhibits EC injury by reducing thrombus formation

**Table 3:** DBMSCs modulate the expression of genes mediating endothelial cell (EC) angiogenesis and migration. THUVEC (HUVEC were cultured with 100 μM H2O2 for 48 h). TDBMSC (HUVEC were cultured with DBMSC and 100 μM H_2_O_2_ for 48 h)

#	Gene symbol	Gene full name	THUVEC mean ΔΔ^−2^ values	TDBMSC mean ΔΔ^−2^ values	Fold change (TDBMSC Vs. THUVEC) *P* < 0.05	Biological activities
1	CAV1	Caveolin-1	12	1472	> 122-fold ↑	Inhibit EC angiogenesis
2	VWF	von Willebrand Factor	4	693	> 173-fold ↑	
3	AGT	Angiotensinogen	1	1018	1018-fold ↑	
4	CASP3	Caspases 3	24	177	> 7-fold ↑	
5	BAX	Bcl-2-associated X	4	27	6.75-fold ↑	
6	F2R	Coagulation Factor II Thrombin Receptor	20	1	20-fold ↓	Induce EC angiogenesis
7	FGF1	Fibroblast Growth Factor 1	3384	0.12	28,200-fold ↓	
8	FGF2	Fibroblast Growth Factor 2	14	4	3.5-fold ↓	
9	KIT	Tyrosine Protein Kinase Kit or CD117	122,521	0.74	> 165568-fold ↓	
10	PTGIS	Prostacyclin Synthase	7	2	3.5-fold ↓	
11	PTGS2	Cyclooxygenase (COX)	15	2	7.5-fold ↓	
12	SELPLG	P-selectin glycoprotein ligand-1	16	4	4-fold ↓	
13	TEK	EK Receptor Tyrosine Kinase (TIE-2)	67	20	3.35-fold ↓	
14	VEGFA	Vascular Endothelial Growth Factor A	2919	0.52	> 5613-fold ↓	
15	CFLAR	CASP8 and FADD like apoptosis regulator	530	85	> 6-fold ↓	
16	EDN1	Endothelin-1	12	82	6.83-fold ↑	Induce EC migration
17	SPHK1	Sphingosine Kinase 1	44	840,958	19112.68-fold ↑	
18	HMOX1	Heme Oxygenase-1	7	158	22.57-fold ↑

**Table 4:** DBMSCs modulate the expression of genes mediating endothelial cell (EC) permeability. THUVEC (HUVEC were cultured with 100 μM H2O2 for 48 h). TDBMSC (HUVEC were cultured with DBMSC and 100 μM H_2_O_2_ for 48 h)

#	Gene Symbol	Gene full name	THUVEC Mean ΔΔ^−2^ values	TDBMSC Mean ΔΔ^−2^ values	Fold change (TDBMSC Vs. THUVEC) *P* < 0.05	Biological activities
1	ACE	Angiotensin I Converting Enzyme	15,765	5	3153-fold ↓	Induce EC permeability
2	ADAM17	A disintegrin and Metalloprotease 17	256	4	64-fold ↓	
3	IL1β	Interleukin 1 beta	4	0.46	8.69-fold ↓	
4	IL6	Interleukin 6	194	59	> 3-fold ↓	
5	VEGFA	Vascular Endothelial Growth Factor A	2919	0.52	> 5613-fold ↓	
6	CAV1	Caveolin-1	12	1472	> 122-fold ↑	Inhibit EC permeability
7	NPR1	Natriuretic Peptide Receptor A/ Guanylate Cyclase A (Atrionatriuretic Peptide Receptor A)	1	12	12-fold ↑

**Table 5:** DBMSCs modulate the expression of genes mediating leukocyte infiltration of endothelial cells (EC), adhesion of inflammatory cells and monocyte adhesion and transmigration. THUVEC (HUVEC were cultured with 100 μM H2O2 for 48 h). TDBMSC (HUVEC were cultured with DBMSC and 100 μM H_2_O_2_ for 48 h)

#	Gene symbol	Gene full name	THUVEC mean ΔΔ^−2^ values	TDBMSC mean ΔΔ^−2^ values	Fold change (TDBMSC Vs. THUVEC) *P* < 0.05	Biological activities
1	CDH5	VE-Cadherin (Vascular Endothelial Cadherin)	61	3	~ 20-fold ↓	Induce leukocyte infiltration
2	SELE	E- selectin	21	1	21-fold ↓	
3	VCAM1	Vascular Cell Adhesion Molecule 1	7	0.26	26.92-fold ↓	
4	PLG	Plasminogen	8	90	~ 11-fold ↑	Inhibits adhesion of inflammatory cells
5	THBS1	Thrombospondin 1 (TSP-1)	1097	3	> 365-fold ↓	Induces monocyte adhesion and transmigration

**Table 6:** DBMSCs effects on genes involved in endothelial cell (EC) biology. THUVEC (HUVEC were cultured with 100 μM H2O2 for 48 h). TDBMSC (HUVEC were cultured with DBMSC and 100 μM H_2_O_2_ for 48 h)

#	Gene symbol	Gene full name	THUVEC mean ΔΔ^−2^ values	TDBMSC mean ΔΔ^−2^ values	Fold change (TDBMSC Vs. THUVEC) *P* < 0.05
1	ANGPT1	Angiopoietin 1	1.35	0.64	Fold change is not statically significant, *P* > 0.05
2	AGTR1	Angiotensin II Receptor Type 1	0.13	0.05	
3	ALOX5	Arachidonate 5-Lipoxygenase	0.61	0.36	
4	ANXA5	Annexin A5	9	5	
5	APOE	Apolipoprotein E	0.01	0.01	
6	BCL2L1	BCL2L1	11	10	
7	CALCA	Calcitonin Related Polypeptide Alpha	0.71	0.70	
8	CCL2	C-C motif chemokine ligand 2	1	0.39	
9	CX3CL1	C-X3-C Motif Chemokine Ligand 1	0.50	0.34	
10	EDN2	Endothelin 2	0.01	0.01	
11	FASLG	Fas Ligand	0.39	2.54	
12	FN1	Fibronectin 1	0.06	0.04	
13	ICAM1	Intercellular Adhesion Molecule 1	1	0.30	
14	IL3	Interleukin 3	0.66	1.47	
15	IL7	Interleukin 7	0.10	2.81	
16	KLK3	Kallikrein Related Peptidase 3	1	2	
17	MMP1	Matrix Metallopeptidase 1	3	3	
18	NOS3	Nitric Oxide Synthase 3	1	0.92	
19	NPPB	Natriuretic Peptide B	0.80	0.32	
20	OCLN	Occludin	4.99	1.48	
21	PDGFRA	Platelet Derived Growth Factor Receptor Alpha	1.06	0.26	
22	PGF	Placental Growth Factor	1.40	2.96	
23	PLAT	Plasminogen Activator, Tissue Type	3.73	0.34	
24	PLAU	Plasminogen Activator, Urokinase	1.59	2	
25	SELL	Selectin L	0.16	0.09	
26	SOD1	Superoxide Dismutase 1	17	17	
27	TGFB1	Transforming Growth Factor Beta 1	9	7	
28	ENG	Endoglin	19	16	

## Discussion

We previously reported that DBMSCs protect endothelial cell activation by reducing the adhesion of monocytes to endothelial cells and their stimulatory effect on the proliferation of endothelial cells [2, 12]. These two events are the basis of endothelial cell injury in inflammatory diseases, such as atherosclerosis [12]. Inflammatory diseases are also associated with high level of oxidative stress mediators, such as H_2_O_2_ [15–19]. Recently, we reported the ability of DBMSCs to survive and function under the stress of H_2_O_2_ [20]. In addition, DBMSCs inhibit the angiogenesis of endothelial cells in H_2_O_2_ environment [20]. Therefore, DBMSCs have the potential to be used as a cell-based therapy for the treatment of inflammatory diseases. In this study, we investigated the ability of DBMSCs to protect endothelial cell functions from stress induced by both H_2_O_2_ and monocytes.

First, we determined the effect of DBMSCs on endothelial cell function under H_2_O_2_. DBMSCs significantly induced the stimulatory effect of H_2_O_2_ on endothelial cell proliferation (Fig. 3a, b). This contrasts with our recent finding that MSCs from the chorionic villi of human placentae (pMSCs) reverse the proliferative effect of H_2_O_2_ on endothelial cells [13]. This discrepancy can be attributed to the niche of both DBMSCs and pMSCs. During normal pregnancy, DBMSCs are located in the decidua where they are in a continuous exposure to high levels of oxidative stress mediators, because they are in a closed proximity to the maternal vessels [21, 22] while pMSCs are usually exposed to a lower levels of oxidative stress mediators because they are in a continuous contact with the fetal circulation [6, 7]. Therefore, DBMSCs have possibly acquired characteristics similar to H_2_O_2_ on the functions of endothelial cells.

Next, we demonstrated that the stimulatory effects of DBMSCs and H_2_O_2_ on endothelial cell proliferation are reversible (Fig. 4). However, the paracrine communication between DBMSCs and endothelial cells in the presence of H_2_O_2_ showed more stimulatory effect on endothelial cell proliferation than CMDBMSC (molecules produced by unstimulated DBMSCs) and ICDBMSC (intercellular direct contact), Fig. 4b, c. We also showed that DBMSCs may protect endothelial cells from oxidative stress by the finding that DBMSCs can induce the expression of many genes mediating the survival of endothelial cells and can also reduce the expression of genes that trigger apoptosis, injury, and inflammation in endothelial cells (Table 2) [23–48]. This protective role for DBMSCs on endothelial cells from stress induced by H_2_O_2_ is further confirmed by the ability of DBMSCs to increase the activities of glutathione and thioredoxin reductases (antioxidant enzymes) in H_2_O_2_-treated endothelial cells. Therefore, DBMSCs can protect endothelial cells from stress induced by H_2_O_2_, therefore suggesting a therapeutic potential for DBMSCs in inflammatory diseases.

We also found that ICDBMSC can reduce the stimulatory effect of H_2_O_2_ on endothelial cell adhesion (Fig. 5). In contrast, CMDBMSC and SFDBMSC could not reverse the stimulatory effect of H_2_O_2_ on endothelial cell adhesion (Fig. 5). Instead, SFDBMSC induced the stimulatory effect of H_2_O_2_ on the adhesiveness of endothelial cells. H_2_O_2_ is known to increase the adhesiveness property of endothelial cells through ICAM-1 and VCAM-1 [49–52]. DBMSCs secrete IL-1β and IL-10 [2]. IL-1β is a proinflammatory cytokine that induces endothelial cell production of H_2_O_2_ while IL-10 is an anti-inflammatory cytokine that reduces endothelial cell production of H_2_O_2_ [53, 54]. This may explain why DBMSCs exert dual functions on endothelial cell adhesion depending on the nature of DBMSC treatment. Therefore, paracrine communication with endothelial cells may stimulate DBMSC production of IL1-β while the intercellular direct contact with endothelial cells may stimulate DBMSC production of IL-10. The interaction with DBMSCs (ICDBMSC) reduced endothelial cell expression of VCAM (Table 5), thus suggesting that VCAM may mediate the anti-adhesive effect of DBMSC on endothelial cells. Our data highlight that DBMSCs have dual effects “a double-edged sword” on endothelial cells as it was previously reported for the immunomodulatory properties of bone marrow-derived MSCs [55]. However, a future study is essential to reveal this mechanism.

DBMSCs show also dual effects on endothelial cell migration. DBMSCs (SFDBMSC and ICDBMSC) reversed the inhibitory effect of H_2_O_2_ on endothelial cell migration while CMDBMSC enhanced the inhibitory effect of H_2_O_2_ on the migration of endothelial cells (Fig. 6). In this study, we found that DBMSCs induced the expression of a number of genes (e.g., endothelin-1, sphingosine kinase 1, and heme oxygenase-1) by H_2_O_2_-treated endothelial cells. These genes mediate the migration of endothelial cells [56–58], thus suggesting that these genes may mediate the stimulatory effect of DBMSCs on endothelial cell migration.

Adhesion and migration are the early steps towards endothelial cell angiogenesis [12]. Recently, we showed that DBMSCs inhibit H_2_O_2_-treated endothelial cell angiogenesis [20]. In this study, we found that DBMSCs increased and decreased H_2_O_2_-treated endothelial cell expression of various antiangiogenic [25, 59–63] and proangiogenic [24, 39, 64–70] genes, respectively (Table 3). These data suggest that these genes may mediate DBMSC inhibitory effect on endothelial cell angiogenesis. However, future functional studies are essential to elucidate the roles of these genes in the antiangiogenic properties of DBMSCs.

We previously reported that DBMSCs have an inhibitory effect on monocyte induction of endothelial cell proliferation and on their adhesion to endothelial cells [12]. In this study, we also show that in the presence of H_2_O_2_, DBMSCs inhibit the adhesion of monocytes to endothelial cells (Fig. 8) and also inhibit endothelial cell proliferation (Fig. 7b). This further confirms the protective role of DBMSCs on endothelial cells proliferation (discussed above) from oxidative stress. These data indicate that DBMSCs have the ability to reduced endothelial cell proliferation, a pathological phenomenon that is known to contribute to the formation of atheroma plaque in atherosclerosis [71].

DBMSCs reduced monocyte expression of VCAM-1 (Fig. 9b), thus indicating that this adhesion molecule may mediate monocyte adhesion to endothelial cells. We also found that DBMSCs modulated H_2_O_2_-treated endothelial cell expression of various genes mediating endothelial cell proliferation, adhesion [44, 45], and permeability [38, 40, 59, 69, 72–74] as well as monocyte infiltration of endothelial cells [44, 75, 76]. Together, these data demonstrate the protective roles that DBMSCs may exert on endothelial cells via mechanisms may involve genes listed in Tables 2, 3, 4, and 5. However, functional studies are necessary to elucidate the effects of these genes in mediating DBMCs protective activities on endothelial cells.

## Conclusions

This is the first comprehensive study to demonstrate the protective role of DBMSCs on endothelial cells in harsh oxidative stress environment. DBMSCs can protect endothelial cells from injury induced by oxidative stress or immune cells. Endothelial cell injury is a hallmark of inflammatory diseases, such as atherosclerosis where endothelial cells show increased functional activities (proliferation, adhesion, migration, angiogenesis, and permeability) and increased fibrin and thrombus formation as well as increased adhesion to immune cells, such as monocytes and their infiltration. These functional activities could be therapeutical targets for DBMSCs to repair endothelial cell injury and treat atherosclerosis.
